# 361. Aseptic Meningitis Associated to SARS Cov2 Infection and MIS-C: Pediatric Presentation of Covid-19

**DOI:** 10.1093/ofid/ofab466.562

**Published:** 2021-12-04

**Authors:** Mónica J Olguín Quintero, Sergio RenÉ Bonilla Pellegrini, Rodolfo N JimÉnez JuÁrez, María Citlalli Casillas Casillas

**Affiliations:** 1 Hospital Infantil de México Federico Gómez, Ciudad de México, Mexico City, Mexico; 2 Hospital Infantil de México, Ciudad de México, Distrito Federal, Mexico; 3 Hospital Español, Miguel Hidalgo, Distrito Federal, Mexico

## Abstract

**Background:**

Novel SARS CoV2 may target the central nervous system and several neurological symptoms have been reported in patients with Coronavirus disease (COVID-19). Mucocutaneous and inflammatory symptoms are important in pediatric population associated to immune dysregulation. There are few reports of clinical manifestations in children and less frequently the isolation and affection of Central Nervous System.

**Methods:**

A previously healthy four months female infant with familiar contact to SARS-CoV2 four weeks ago. Start with fever of 104°F, vomiting, maculopapular rash on the anterior thorax and upper extremities involving the palms and soles associated with edema. On physical examination, irritable, bulging anterior fontanelle, non-purulent bilateral conjunctival injection, cheilitis and rash was confirmed.

**Results:**

Laboratory findings: thrombocytopenia, elevated D-Dimer, fibrinogen, PCT, CRP, ferritin and ESR with hypoalbuminemia. MIS-C is integrated with cutaneous, gastrointestinal and neurological affections. Empirically ceftriaxone, vancomycin and acyclovir are started due to suspicion of meningoencephalitis. RT-PCR for SARS-CoV-2 positive. CSF: transparent appearance, slightly xanthochromic color, coagulation and negative film. Proteins 105 mg / dl, glucose 45 mg / dl, leukocytes 121 mm3, erythrocytes 66 mm3, PMN 8% and MNN 92%. Negative culture,PCR Herpes Virus negative,Viral load for SARS CoV2 in CSF 3,400 cop / ml and plasma 118,900 cop / ml, Aseptic meningitis is confirmed by SARS-CoV-2. Antiviral and antibiotics are discontinued and Gamma globulin and methylprednisolone are administered. Evolving favorably and egress at 6th day to complete oral steroid treatment for 3 more day.

**Conclusion:**

The mechanism by which SARS-CoV2 affects the CNS is still unknown.This findings suggests direct infection can be possible. Although it is also described vascular affection has been found that the Spike protein of the virus binds to ACE-2 receptor present in the cerebral vascular endothelium. Neurological manifestations have been described even without respiratory symptoms. A novel pediatric case with viral load for SARS-CoV-2 in CSF is demonstrated. Importance of detecting SARS-CoV-2 in children with encephalitis, which can progress satisfactorily.

**Disclosures:**

**All Authors**: No reported disclosures

